# EXPLORING THE INTERSECTIONALITY OF PLACE AND GENDER AMONG OLDER ADULTS IN GHANA

**DOI:** 10.1093/geroni/igae098.1715

**Published:** 2024-12-31

**Authors:** Shane Burns, Latrica Best, Solomon Amoatey

**Affiliations:** University of Michigan, Ann Arbor, Michigan, United States; Boston College, Chestnut Hill, Massachusetts, United States; Louisville University, Louisville, Kentucky, United States

## Abstract

Ghana’s older adult population is growing rapidly and projected to double by 2050. It is well-documented that social, health, and housing factors influence disparate rates of disability. However, little is known how the intersection of place (i.e., urban; rural) and gender (i.e., woman; man) inform rates of disability among older Ghanaians. We seek to examine this gap in the literature through an intersectional approach. Using logistic regression with Wave 1 (2007/08) data from the Study on global AGEing and adult health (SAGE) Ghana, we investigate the prevalence of activities of daily living (ADL) disability among respondents ages 50+ (n=4,106). To document gender differences by place, we compute separate adjusted odds ratio models among urban and rural respondents while accounting for health, social, and housing factors that might explain gender differences. Compared to urban men, urban women’s ADL disability disadvantage was explained by marital status, particularly widowhood. In contrast, rural women consistently reported an ADL disability disadvantage when compared to rural men. Additionally, we found that the morbidity profiles of those who reported ADL disability differed by place and certain ADL difficulties (i.e., bed transferring; toileting) were especially common among women. Women, both urban and rural, were especially vulnerable to ADL disability. Marital status explaining urban women’s ADL disability disadvantage suggests that partnership attenuates disability risk. Also, we speculate that varied morbidity associations with ADL disability are due to different stressors in urban versus rural environments. These findings also generate further interest about rural women’s disability disadvantage.

